# Beta-thalassemia trait: an underrecognized risk for osteoporosis in postmenopausal women, warranting screening

**DOI:** 10.1530/EDM-25-0073

**Published:** 2025-09-11

**Authors:** Pooja Alipuria, Atush Alipuria

**Affiliations:** ^1^ Department of Internal Medicine, Yashoda Super Speciality Hospital, Ghaziabad, India; ^2^ Department of Pulmonary Medicine, Yashoda Super Speciality Hospital, Ghaziabad, India

**Keywords:** aging, beta-thalassemia trait, osteoporosis, bone inflammation, bone health

## Abstract

**Summary:**

This case series presents two postmenopausal women with beta-thalassemia trait who developed osteoporosis. Case 1 involves a woman in her 70s presenting with persistent lower back pain; imaging revealed a compression fracture at L2, and a DEXA scan confirmed osteoporosis with a forearm T-score of −3.8 and a femoral neck T-score of −2.5. Case 2 describes a woman in her late 50s with generalized bone pain and severe osteoporosis identified through DEXA scanning, with a lumbar spine T-score of −3.3. Both patients lacked classical secondary causes of bone loss, and laboratory evaluations were unremarkable. Family history was notable for osteoporosis in first-degree relatives, though the relatives’ thalassemia status was unknown. Both patients declined injectable therapies and were managed with oral alendronate, calcium, and vitamin D supplementation. These cases highlight beta-thalassemia trait as a potential underrecognized risk factor for osteoporosis in postmenopausal women, suggesting the need for further research and consideration in clinical guidelines.

**Learning points:**

- Beta-thalassemia trait may predispose to osteoporosis, even without iron overload or transfusion dependence.
- Postmenopausal women with beta-thalassemia trait should undergo early DEXA screening to prevent fractures.
- Patient preference impacts management: oral bisphosphonates are viable when injectables are refused.
- Guideline gaps: current osteoporosis protocols do not address beta-thalassemia trait as a risk factor.

## Background

This case report is significant as it highlights a potential underrecognized association between beta-thalassemia trait and osteoporosis in postmenopausal women. While osteoporosis is a well-documented complication in beta-thalassemia major and intermedia, primarily due to factors such as iron overload and bone marrow expansion, its occurrence in individuals with beta-thalassemia minor – who typically do not require transfusions and lack iron overload – is less understood. Emerging evidence suggests that beta-thalassemia minor may independently contribute to decreased bone mineral density, possibly through mechanisms such as ineffective erythropoiesis and hormonal imbalances. Despite these findings, current osteoporosis screening guidelines do not specifically address beta-thalassemia trait as a risk factor. By presenting two cases of postmenopausal women with beta-thalassemia trait who developed osteoporosis without other common risk factors, this report underscores the need for increased awareness and consideration of beta-thalassemia minor in osteoporosis risk assessments. It also highlights the importance of individualized patient management, particularly when standard treatments are declined, and calls for further research to inform clinical guidelines.

## Case presentation

### Case 1

A postmenopausal woman (attained menopause at 52 years) in her 70s with a known beta-thalassemia trait presented in early 2025 with persistent lower back pain. MRI of the lumbosacral spine revealed a compression fracture of the L2 vertebral body with significant retropulsion of the posterior vertebral wall into the spinal canal, leading to compression of the cauda equina nerve roots. In addition, broad-based disc bulges at the L2–L5 levels caused thecal sac indentation and mild bilateral neural foraminal narrowing. These findings correlated with her clinical symptoms and were significant for potential neurological compromise. A DEXA scan performed in January 2025 demonstrated osteoporosis, with T-scores of −2.4 at the lumbar spine, −2.5 at the femoral neck, −2.2 at the left total femur, and −3.8 at the left forearm.

The patient has no known comorbidities. Family history is significant for severe osteoporosis in her mother, although the history of fractures and thalassemia status is not known.

### Case 2

A postmenopausal woman (attained menopause at 51 years) in her late 50s with a known beta-thalassemia trait presented with generalized bone pain. A DEXA scan revealed severe osteoporosis, with T-scores of −3.3 at the lumbar spine, −2.9 at the left femoral neck, −3.0 at the left total femur, and −2.7 for total body. The Z-score at L1–L4 was −2.0.

The patient has no known comorbidities. Family history is significant for osteoporosis in her sister, who is currently on medication for the condition; the sister’s thalassemia status is not known.

## Investigation

### Case 1

#### Laboratory evaluations

- Serum tests: normal serum calcium, phosphate, alkaline phosphatase, vitamin D, and renal and liver function tests.
- Intact parathyroid hormone (PTH) levels, serum protein electrophoresis (SPEP) with immunofixation, and serum free light chain assays were performed to exclude hyperparathyroidism and plasma cell dyscrasias; all results were within normal limits.
- Inflammatory markers: erythrocyte sedimentation rate (ESR) was normal.
- Gastrointestinal evaluation: stool occult blood tests were negative, and there were no signs or symptoms suggestive of malabsorption.
- Thyroid function tests: thyroid-stimulating hormone (TSH): initial 5.6 mIU/L → repeat 4.9 mIU/L → subsequent 3.66 mIU/L (normal range: 0.4–4.2). Anti-TPO antibodies: 359 IU/mL (positive; normal <34). Free T3/T4: within normal limits.

#### Hematological findings

- Complete blood count (CBC): hemoglobin (Hb) 9.5 g/dL, mean corpuscular volume (MCV) 60.3 fL, mean corpuscular hemoglobin (MCH) 18.1 pg, red cell distribution width (RDW) 19.2%.
- Peripheral smear: microcytic hypochromic anemia.
- Iron studies: serum ferritin 31.4 ng/mL, transferrin saturation (TSAT) 10.95%, serum iron 40 μg/dL, total iron-binding capacity (TIBC) 365 μg/dL.
- Folic acid: 11 ng/mL.

#### Interpretation

The hematological profile indicates microcytic hypochromic anemia with elevated RDW and low TSAT, suggestive of concurrent iron deficiency anemia. Given the known beta-thalassemia trait, a dual pathology is likely, necessitating a comprehensive management approach.

### Case 2

#### Laboratory evaluations

- Serum tests: normal serum calcium, phosphate, alkaline phosphatase, and renal and liver function tests.
- Thyroid function: TSH was mildly elevated at 6.94 mIU/L, with normal free T3 (FT3) and free T4 (FT4), consistent with subclinical hypothyroidism. Anti-thyroid peroxidase (anti-TPO) antibodies were negative.
- Vitamin D: serum 25-hydroxyvitamin D was insufficient at presentation but was subsequently corrected with weekly cholecalciferol supplementation (60,000 IU), leading to normalization of vitamin D levels.
- Further evaluations – including intact parathyroid hormone (PTH) levels and serum protein electrophoresis (SPEP) – were recommended to exclude hyperparathyroidism and plasma cell dyscrasias. However, these tests were deferred due to the patient’s preference.
- Inflammatory markers: ESR was within normal limits.

#### Hematological findings

- CBC: hemoglobin (Hb) 9.4 g/dL, red blood cell (RBC) count 5.6 million/μL, packed cell volume 30.4%, MCV 54.6 fL, MCH 17 pg, MCHC 31 g/dL, RDW-CV 17%, total leukocyte count 9,000/μL, and platelet count 322,000/μL.
- Mentzer index: 9.75.

#### Interpretation

The low MCV and MCH, coupled with a high RBC count and a Mentzer index below 13, are indicative of beta-thalassemia trait. The anemia observed may be primarily due to thalassemia, with no significant evidence of iron deficiency.

## Treatment

Both patients were advised injectable therapies (teriparatide or denosumab) but declined due to personal preference, opting instead for oral alendronate 70 mg once weekly. Calcium and vitamin D supplementation were provided as per guideline recommendations to support bone health. In the first case, oral iron therapy was prescribed for iron deficiency anemia, though adherence remained inconsistent. These cases highlight patient-driven preferences for oral regimens over injectables, emphasizing the need for personalized therapeutic strategies to optimize adherence and outcomes.

## Outcome and follow-up

### Case 1

At follow-up, the patient reported some improvement in symptoms, although back pain persisted. Pain management specialists recommended surgical intervention, but the patient opted against surgery. Plans were made to assess bone turnover markers (e.g. serum P1NP and CTx) after 3 months to monitor treatment response, and to repeat DEXA scanning after 2–3 years, in line with clinical guidelines. Given the patient’s inconsistent adherence to iron supplementation, counseling on the importance of regular intake was provided, and strategies to improve compliance were discussed.

### Case 2

At follow-up, the patient reported symptomatic improvement. Plans were made to assess bone turnover markers (e.g. serum P1NP and CTx) after 3 months to monitor treatment response, and to repeat DEXA scanning after 2–3 years, in line with clinical guidelines.

## Discussion

### Clinical implications and screening considerations

Beta-thalassemia minor (βTT), traditionally dismissed as a benign carrier state, may harbor underrecognized skeletal risks in postmenopausal women. Our cases – two postmenopausal βTT patients with severe osteoporosis (T-scores ≤−3.3) and no traditional risk factors – highlight a potential gap in current osteoporosis guidelines (e.g., NOF) (1). While limited to two cases, these findings suggest βTT may compound age-related bone loss, warranting further exploration of its role as a secondary risk factor. Notably, both patients declined injectable therapies (denosumab/teriparatide) in favor of oral bisphosphonates, underscoring the importance of patient preference in adherence – a factor critical to therapeutic success (1).

### Mechanistic hypotheses: bridging hematology and bone biology

#### Bone marrow dynamics

Compensatory erythropoiesis in βTT may drive low-grade marrow hyperplasia, potentially encroaching on trabecular architecture and disrupting bone remodeling (2). While subtle, this expansion – evidenced by inverse marrow cellularity-BMD correlations in hematopoietic disorders (2) – could synergize with postmenopausal estrogen deficiency, crippling compensatory bone formation (3).

#### Genetic susceptibility

Polymorphisms in *COL1A1* (collagen type I alpha 1) and *VDR* (vitamin D receptor), implicated in osteoporosis among beta-thalassemia major (β-TM) patients (4), may also predispose beta-thalassemia minor (βTT) carriers to skeletal fragility. For instance, the *VDR* Fok1 CC genotype, linked to reduced lumbar BMD in β-TM (4), and familial osteoporosis histories in our βTT cases suggest a shared genetic vulnerability across thalassemia syndromes. This interplay – potentially involving iron overload, collagen defects, or vitamin D dysregulation – warrants investigation in larger βTT cohorts to clarify its role in bone health deterioration.

#### Inflammation and oxidative stress

Chronic low-grade inflammation in βTT could elevate pro-resorptive cytokines (IL-6, TNF-α), and reactive oxygen species, fostering a pro-osteoclastic milieu (3, 4). Unlike β-TM, iron overload is unlikely here; instead, subclinical erythroid stress might perpetuate bone loss, amplified by age-related oxidative damage (4).

### A roadmap for future research

While our observations are preliminary, they propose βTT as a potential modifier of postmenopausal osteoporosis risk. We urge:

- Prospective studies to quantify osteoporosis prevalence in βTT women ≥50 years.
- Genetic analyses to explore *COL1A1/VDR* polymorphisms in βTT cohorts with low BMD.
- Mechanistic investigations into marrow expansion and cytokine profiles in βTT.

## Conclusion

βTT exemplifies the intersection of hematologic and skeletal pathophysiology – a duality demanding rigorous inquiry. Our cases, while anecdotal, challenge complacency and propose βTT as a candidate risk modifier for osteoporosis. Until larger studies validate this association and influence the guidelines, clinicians might consider DEXA screening in postmenopausal βTT women with additional risks (e.g. familial osteoporosis). Future research should quantify osteoporosis risk in beta-thalassemia trait, explore severity gradients across thalassemia syndromes, and clarify underlying mechanisms. Let these findings ignite curiosity, not certainty, and catalyze research into this uncharted nexus.

## Declaration of interest

The authors declare that there is no conflict of interest that could be perceived as prejudicing the impartiality of the research reported.

## Funding

This research did not receive any specific grant from any funding agency in the public, commercial, or not-for-profit sector.

## Patient consent

Written informed consent for publication of their clinical details and clinical images was obtained from the patient.

## Author contribution statement

PA and AA contributed equally to the conception and design of the case report. PA oversaw the patient’s clinical management and drafted the initial manuscript, while AA contributed to the analysis of the clinical data and critically revised the manuscript. Both authors reviewed and approved the final manuscript.
